# Troglitazone Reduces Glyoxalase I Protein Expression in Glioma and Potentiates the Effects of Chemotherapeutic Agents

**DOI:** 10.1155/2010/373491

**Published:** 2010-05-04

**Authors:** Jeffrey Helgager, Jie Li, Irina A. Lubensky, Russell Lonser, Zhengping Zhuang

**Affiliations:** ^1^Surgical Neurology Branch, National Institute of Neurological Disorders and Stroke, National Cancer Institute, National Institutes of Health, Bethesda, MD 20892-1414, USA; ^2^Cancer Diagnosis Program, Division of Cancer Treatment and Diagnosis, National Cancer Institute, National Institutes of Health, Bethesda, MD 20892-1414, USA

## Abstract

Despite resistance of most gliomas to chemotherapy, approximately 2/3 of oligodendrogliomas show sensitivity to such agents. This sensitivity has been associated with deletions on chromosome 1p alone or in combination with 19q. Higher expression of the enzyme glyoxalase I has been found in oligodendrogliomas with chromosome 1p intact compared to those with a deletion. Higher expression of this enzyme is also associated with tumor chemoresistance in other cancers. The present study tested whether the drug troglitazone would make a glioma cell line more sensitive to chemotherapeutic agents. This drug was chosen because it has been shown to decrease glyoxalase I enzyme activity in cells. Treatment with troglitazone decreased expression of glyoxalase I, and potentiated cell death when used in combination with chemotherapeutic agents. This decrease in glyoxalase I protein may be one mechanism by which this potentiation occurs, and troglitazone may be a candidate for use in glioma therapy.

## 1. Introduction

Gliomas are the most common primary CNS malignancy, with more than 12,500 diagnosed annually in the U.S. These tumors carry a dismal prognosis despite all treatment efforts, with a median survival of 9–15 months for the most common and highest grade, glioblastoma multiforme (GBM), and even low-grade tumors usually resulting in a fatal outcome. Although a meager survival benefit has been shown with chemotherapy, as a whole these malignancies are very resistant to such treatments. An exception is found in oligodendrogliomas, approximately 15% of all gliomas diagnosed, in which about 2/3 of cases show dramatic responses to chemotherapeutic agents [1]. These enhanced responses have been associated with allelic losses on chromosomes 1p either alone or in combination with 19q. Patients with tumors harboring such deletions fare dramatically better than those with tumors that have the chromosomes intact [2]. As of present, however, the precise mechanisms responsible for this susceptibility have not been identified [1].

We previously demonstrated significantly higher expression of the enzyme glyoxalase I (GLO-1) in human oligodendroglioma tumor specimens with chromosome 1p intact compared to those with 1p losses [3]. GLO-1 is a cytosolic protein that functions as the major detoxifier of *α*-oxoaldehydes in cells, predominantly methylglyoxal. Methylglyoxal is an obligatory byproduct of glycolysis, and may be produced in greater quantities with activation of DNA repair processes, induced by chemotherapeutic agents in response to their mutagenic effects. Methylglyoxal and other *α*-oxoaldehydes glycate DNA, RNA, and proteins, forming advanced glycation end products (AGEs) [4]. These covalent modifications of DNA lead to mutations, and glycated proteins are degraded [5]. Probably through these mechanisms, methylglyoxal is capable of inducing apoptosis in tumor cells [4]. 

This enzyme has been shown to contribute to the resistance of tumors to chemotherapeutic agents. Over-expressing GLO-1 in leukemia cells conferred resistance to chemotherapeutic drugs, whereas decreasing expression in lung cancer cells expressing high levels of the enzyme induced chemosensitivity [6, 7]. A synthetic inhibitor of GLO-1 sensitized chemoresistant leukemia cells over-expressing the enzyme to chemotherapy, and this compound has shown significant antiproliferative effects when used on tissue xenografts in small animals [4]. Because of the association of lower GLO-1 expression in oligodendrogliomas with chromosome 1p losses, and the notable role of this enzyme in cancer chemoresistance, decreasing expression of GLO-1 in gliomas expressing it at high levels may make them more susceptible to chemotherapy. Interestingly, GLO-1 is located on chromosome 6, so the 1p loss in these chemosensitive oligodendrogliomas does not directly disrupt the GLO-1 gene. We speculate that one or more genes disrupted on chromosome 1p positively regulate GLO-1 expression through an as yet unknown mechanism [3].

Troglitazone (TRG) is an insulin-sensitizing agent that has been used for the treatment of type II diabetes, and is in the thiazolidinedione class of drugs that exert their therapeutic effects through agonism of the peroxisome proliferator-activated receptor-*γ* (PPAR*γ*), a nuclear receptor which regulates the transcription of a variety of downstream gene targets. In addition to its insulin sensitizing actions, TRG and another compound in its class, ciglitazone, are unique among the thiazolidinediones in having the ability to exert antiproliferative effects against many cancer cell lines. Although the mechanisms remain elusive, these agents have been shown to induce apoptosis in malignant cells, and data support that this effect is independent of the PPAR*γ* agonism responsible for the insulin-sensitizing properties of these drugs [8]. TRG has been found to decrease the enzymatic activity of GLO-1 and transcription of its mRNA in both normal and malignant cells. This effect is also most likely PPAR*γ* independent because another PPAR*γ* agonist, rosiglitazone, did not influence GLO-1 expression [9]. When used in conjunction with doxorubicin (DOX), troglitazone was shown to cause synergistic cell death in a resistant leukemia cell line that expressed high levels of GLO-1 [10]. 

In order to investigate the anticancer properties of TRG in glioma, we used the astrocytoma cell line U-373 as an in vitro model of this malignancy. We examined the impact of TRG on GLO-1 expression in these cells, and the tumoricidal effects of combining this drug with chemotherapeutic agents. Our findings have potential clinical implications for treating these malignancies.

## 2. Materials and Methods

### 2.1. Drug Treatments

Previously established U-373, U-87, A-172, and U-251 human glioma cells were obtained from ATCC (Manassas, VA) and grown in Dulbecco's Modified Eagle Medium supplemented with 10% FCS. TRG, DOX, and carmustine (BCNU) were obtained from Sigma-Aldrich Co. (St. Louis, MO). TRG was dissolved in DMSO, DOX in molecular-grade water, and BCNU in 100% ethanol. Drug treatments were performed one time at the beginning of the course of observation. U-373 cells were treated with varying concentrations of TRG: 1, 5, 10, 25, 50, and 100 *μ*M. The final doses of chemotherapeutic drugs for combination treatments with TRG were optimized according to applicable clinical dosages so as to achieve only moderate cytotoxicity. Concentrations of 50, 100, 150, 200, and 500 nM for DOX and 100, 200, 300, 400, 500, and 1000 *μ*M concentrations for BCNU were investigated. The optimized concentrations for DOX and BCNU were determined to be 100 nM and 300 *μ*M, respectively. Cells receiving control treatments had equivalent volumes of the vehicles used for dissolving these drugs added to their media.

### 2.2. Western Blotting

Cells were treated with TRG 24-hours after seeding and harvested in 24 hours time intervals up to the 96-hour time point or when cells were no longer viable. Triplicate experiments were run for each treatment condition. Cell pellets were resuspended in Tissue Protein Extraction Reagent (Pierce, Rockford, IL) and sonicated. Equivalent protein quantities were loaded into Novex 4%–20% Tris-Glycine Gels (Invitrogen, Carlsbad, CA) and separated by electrophoresis. Proteins were then transferred onto nitrocellulose membranes (Millipore Corp., Milford, MA) and incubated with polyclonal antiglyoxalase I (1 : 500; Santa Cruz Biotechnology, Santa Cruz, CA) and polyclonal anti-*β*-actin (1 : 500; Santa Cruz Biotechnology, Santa Cruz, CA) antibodies. Immunosignal was visualized with SuperSignal West Pico Chemiluminescent Substrate (Pierce, Rockford, IL). Densitometry analyses were performed using the program ImageJ, and statistical analysis using Microsoft Excel 2003. GLO-1 protein expression for each sample was normalized to *β*-actin levels for all densitometry analyses.

### 2.3. Semiquantitative Reverse Transcription PCR

Cells were treated with 50 *μ*M TRG 24 hours after seeding and harvested after 24, 48, and 72 hours. Experiments were run in triplicate for each time point. Total RNA was extracted using TRIZOL Reagent (Invitrogen, Carlsbad, CA). Reverse transcription was performed using a SuperScript III First Strand Kit (Invitrogen, Carlsbad, CA) with 1 *μ*g RNA. PCR was done using Platinum PCR SuperMIX High Fidelity (Invitrogen, Carlsbad, CA). GLO-1 primers were obtained from Invitrogen (Carlsbad, CA): 5′-CACTCTACTTCTTGGCTTAT-3′ and 5′-TGTATACATCAGGAACAGCA-3′. The *β*-actin gene was amplified using the following primers: 5′-CCACGAAACTACCTTCAACTCC-3′ and 5′-TCATACTCCTGCTTGCTGATCC-3′. Twenty-seven rounds of a 94°C/30 sec-56°C/30 sec-70°C/30 sec amplification cycle were used in both PCR reactions.

### 2.4. MTT (3-(4, 5-Dimethylthiazolyl-2)-2, 5-Diphenyltetrazolium Bromide) Cell Proliferation Assay

96-well plates were seeded with 1.5 × 10^4^ U-373 cells per well and treated 24-hours after plating. Cells were treated with TRG alone or in combination with DOX or BCNU. Cell viability was measured using the MTT Cell Proliferation Assay (ATCC, Manassas, VA) in 24 hour intervals out to 96 hours following treatment. Colorimetric change was assayed with an absorbance microplate reader at 570 nm. All experiments were run in triplicate with 7-well treatments per group and a two-tailed Student's *t*-test was used for statistical analysis.

### 2.5. Drug Evaluation

Combination drug cytotoxicities as measured by MTT were dubbed as potentiating, additive, or antagonistic. The term potentiation is used to describe a greater than additive effect with combined treatment compared to using either drug alone. In the case of cell cytotoxicity, an additive effect is considered to be the product of the percent cell viabilities when cells are treated with each of the two drugs individually. Therefore, an observed value for combined treatment which is less than additive in a statistically significant fashion (*P* < .05) is considered potentiation. A value which is greater than additive is antagonistic [11].

## 3. Results

### 3.1. GLO-1 Expression in Commercial Glioma Cell Lines

**Figure 1:**
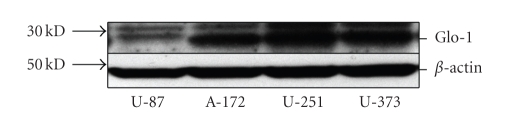
Western blot demonstrating GLO-1 protein expression in four commercial glioma cell lines.

Comparison of GLO-1 expression in the commercially available glioma cell lines U-87, A-172, U-251, and U-373 was assayed by Western blot, yielding a band corresponding to the expected size of 23 kD (Figure 1). Highest GLO-1 expression was detected in U-251 and U-373 cells, with relatively less expression in A-172 and U-87 cells.

### 3.2. TRG Decreases GLO-1 Expression in U-373 Cells

**Figure 2:**
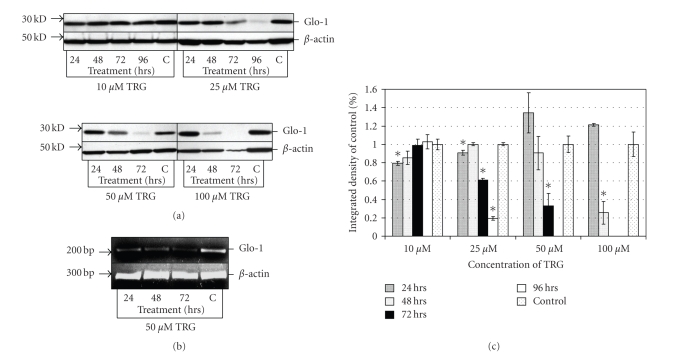
(a) Representative Western blots showing GLO-1 protein expression after treatment of U-373 cells with 10, 25, 50, and 100 *μ*M troglitazone (TRG) for various time periods. Note loss of *β*-actin control at 72 hrs for 100 *μ*M TRG concentration, reflecting loss of cell viability at this time point. (b) A representative gel showing semiquantitative reverse transcription PCR of GLO-1 following treatment of U-373 cells with 50 *μ*M TRG. (c) Densitometry analysis of Western blots showing GLO-1 expression after U-373 cells were treated with concentrations of TRG. Asterisks demark time points with a significance of *P* < .01 compared to control based on Student's two-tailed *t*-test. Each data point is the mean and SEM of three separate experiments performed at the specified condition.

Treatment of U-373 astrocytoma cells with 25, 50, and 100 *μ*M TRG evoked a substantial decrease in the GLO-1 enzyme at the last 24-hour time point before cells were no longer viable for harvesting (Figures 2(a) and 2(c)). This maximal reduction was observed at 48 hours for 100 *μ*M (25.6 ± 1.9% SEM), at 72 hours for 50 *μ*M (32.7 ± 13.6% SEM), and at 96 hours for 25 *μ*M TRG (19.5 ± 12.4% SEM). This decline was not observed even after 96 hours of treatment with 10 *μ*M TRG (102.7 ± 7.7% SEM), the last time point measured. A 204 bp mRNA fragment of GLO-1 mRNA was amplified using RT-PCR, yielding a band of the expected size. Even after 24 hours of treatment with 50 *μ*M TRG, a decline in mRNA expression was observed (Figure 2(b)). There was a further, incremental reduction in mRNA expression seen up to 72 hours after treatment.

### 3.3. Combined Drug Treatment of TRG with DOX or BCNU Potentiates Cell Death

**Figure 3:**
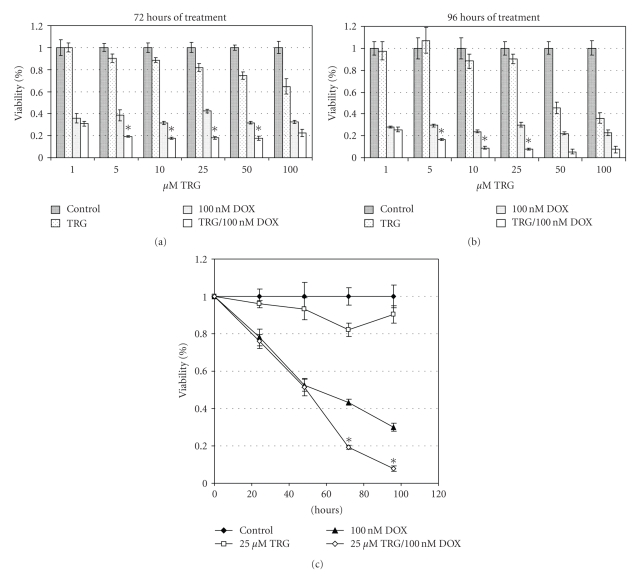
A representative experiment demonstrating U-373 cell viability after 72 (a) and 96 hours (b) of treatment with TRG, 100 nM DOX, and TRG/100 nM DOX together at the specified concentration of TRG. (c) Time course of treatment of U-373 cells with 25 *μ*M troglitazone (TRG), 100 nM doxorubicin (DOX), and TRG/100 nM DOX together. Combination treatments found to potentiate cytotoxicity are marked with an asterisk; other concentrations were found to be additive. Each data point is the mean and SEM of seven wells treated at the specified condition.

Combination treatment with TRG and 100 nM DOX was found to potentiate cell death as measured by the MTT assay when compared to treatments with each drug alone. This phenomenon was observed after three and four days of treatment, occurred at concentrations as low as 5 *μ*M TRG, was greatest at 25 *μ*M, and became progressively less pronounced with higher doses (Figures 3(a) and 3(b)). The latter is probably attributable to TRG treatment alone having a significant tumoricidal effect at these higher concentrations. Cell viability at all time points gathered for 25 *μ*M TRG, where most potentiation was found, is shown (Figure 3(c)). Potentiation was only seen at 72 and 96 hours, not earlier, and this was true for all other concentrations of TRG where potentiation occurred. Greatest potentiation was observed at 96 hours, where combination treatment showed a viability of only 8.0% of control.

**Figure 4:**
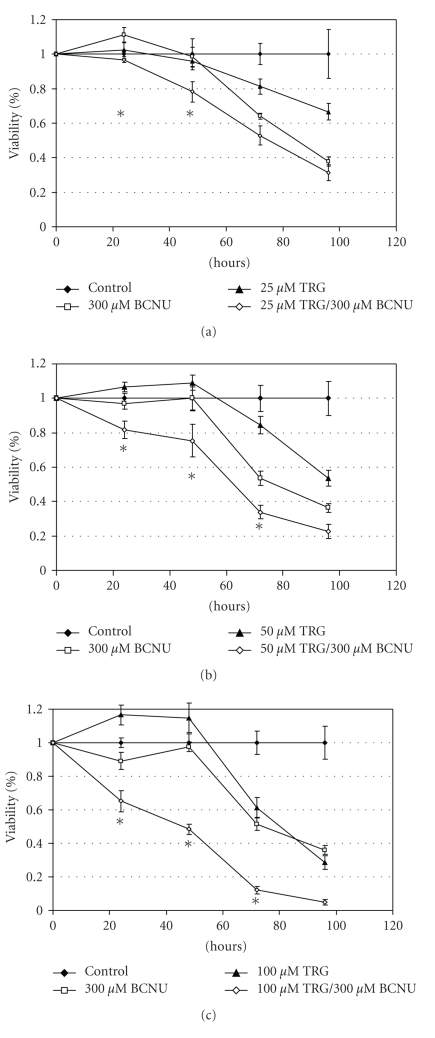
A representative experiment showing cell viability time course with treatment of U-373 cells with TRG, 300 *μ*M BCNU, and TRG/300 *μ*M BCNU together at 25 (a), 50 (b), and 100 (c) *μ*M concentrations of TRG. Combination treatments found to potentiate cytotoxicity are marked with an asterisk; other concentrations were found to be additive. Each data point is the mean and SEM of seven wells treated at the specified condition.

BCNU was also found to potentiate cell death when combined with TRG, but the time points at which this was observed were earlier, and the doses of TRG required for substantial potentiation greater. Though mild potentiation was observed at 25 *μ*M TRG at 24 and 48 hours, the cell viability at 48 hours for combination treatment was only 78.2% of control (Figure 4(a)). For both 50 and 100 *μ*M concentrations of TRG, combination treatment potentiated cell death up to 72 hours, with the most substantial result observed at 100 *μ*M (Figures 4(b) and 4(c)). Though combination treatment had a substantial tumoricidal effect at 96 hours, achieving just 4.8% viability at 100 *μ*M TRG, statistically significant potentiation was not found.

## 4. Discussion

Higher levels of GLO-1 have been found in a broad array of tumors when compared to their normal tissue counterparts including prostate, colon, and renal cancers [12]. This may reflect the increased reliance of tumor cells on anaerobic glycolysis, as well as their intrinsically high metabolic rate; both result in an increased production of methylglyoxal and consequent demand for its detoxification [13]. This suggests that GLO-1 could be a therapeutic target in these more aggressive malignancies, particularly because tumors with higher endogenous activity of this enzyme have been documented to be more susceptible to the cytotoxic effects of its inhibition [12]. 

To test this idea, we assayed GLO-1 protein levels in four select glioma cell lines in order to find one that demonstrated robust expression. Of note, the two cell lines found to express highest levels of GLO-1, U-373, and U-251, have an intact chromosome 1p, whereas U-87 and A-172 have it disrupted [14]. This is consistent with our findings in oligodendrogliomas that GLO-1 expression is higher in malignancies with chromosome 1p intact [3]. However, this relationship has not been studied in other types of glioma and we cannot draw any significant conclusions based on the few cell lines analyzed here. 

We employed the U-373 astrocytoma cell line for all subsequent work. The finding that exposure of these cells to TRG conferred a substantial reduction in GLO-1 is consistent with results obtained elsewhere in other cell types [9]. However, this is the first finding of a decrease in protein expression following treatment with this drug, as GLO-1 enzyme activity was previously assayed instead of amount of protein. Also consistent with these previous findings, concentrations of TRG required to decrease GLO-1 expression were substantially higher than that required to induce full PPAR*γ* agonism, which is around 1 *μ*M [15]. The cytotoxic effects of TRG we observed are therefore unlikely to be PPAR*γ* dependent. 

The reduction in GLO-1 mRNA expression after TRG exposure was observed well before protein expression decreased by Western blot. This suggests that the decrease seen in this enzyme is at least partially mediated by a transcriptional mechanism, also found in other studies employing different cell lines [9, 10]. 

To see whether potentiation of cytotoxicity would occur when TRG was used in combination with chemotherapeutic agents, we first employed the topoisomerase II inhibitor DOX. This drug offers the advantage of being stable in aqueous solution, unlike the alkylating agents employed for glioma therapy, and would be present in the cell culture medium during the period necessary for TRG to exert its drug effect [16, 17]. However, DOX is not generally used for glioma treatment due to poor blood brain barrier penetration, though novel approaches to its administration that circumvent this problem have shown clinical efficacy [18]. A concentration of 100 nM was selected because it conferred moderate cytotoxicity, but is a low clinical dose [19]. In order to examine a more clinically relevant chemotherapeutic drug, we also studied the alkylating agent BCNU, which has been employed as a chemotherapeutic agent against glioma for years. Furthermore, as BCNU works by a similar mechanism of action as temozolomide, the standard chemotherapeutic treatment for glioblastoma, potentiation of the cytotoxic effects of BCNU by TRG suggests that TRG could be similarly efficacious when combined with temozolomide [1]. The concentration of 300 *μ*M BCNU used in these experiments corresponds to a high clinical dose of this drug [20]. 

Significant potentiation of the effect of DOX on cell death was seen at concentrations of TRG as low as 5 *μ*M, which is within clinically achievable concentrations in plasma [21]. This is important, as a major limitation of the tumoricidal thiazolidinediones as anticancer agents has been the inability to demonstrate antiproliferative effects at concentrations which are clinically relevant [8]. Although potentiation of cytotoxicity for BCNU occurred at substantially higher doses of TRG, this finding is notable in light of the lack of efficacious treatments for malignant glioma and the dismal prognosis of this disease.

The time course and doses required for potentiation of cytotoxicity with TRG and both chemotherapeutic agents are not entirely consistent with observed decreases in GLO-1. However, this enzyme is only one of many proteins implicated in contributing to the antiproliferative effects of this drug. Although a decrease in GLO-1 is likely contributing to the observed cytotoxicity with combination treatment, other mechanisms are probably involved. The chemotherapeutic agents used could also have facilitated a fall in GLO-1 enzyme activity that was not observed by Western blot of cells treated with TRG alone. This is particularly relevant because both DOX and BCNU decrease reduced glutathione (GSH) in the cell, a critical cofactor for the functionality of GLO-1 [22]. 

Our data demonstrates that TRG may have future clinical potential in enhancing the effects of chemotherapeutic agents against glioma, and one probable mechanism by which this occurs is through decreasing GLO-1 expression. The results of our experiments suggest that TRG, in addition to other drugs which inhibit the activity of GLO-1, should be looked at further for use in glioma therapy. Through this it may be possible to develop another treatment in the limited armamentarium of drugs against this disease.
